# Phytochemical Screening and Acanthamoebic Activity of Shoots from in Vitro Cultures and in Vivo Plants of *Eryngium alpinum* L.—The Endangered and Protected Species

**DOI:** 10.3390/molecules25061416

**Published:** 2020-03-20

**Authors:** Małgorzata Kikowska, Dariusz Kruszka, Monika Derda, Edward Hadaś, Barbara Thiem

**Affiliations:** 1 Department of Pharmaceutical Botany and Plant Biotechnology, Poznan University of Medical Sciences, 14 Św. Marii Magdaleny St., 61-861 Poznań, Poland; bthiem@ump.edu.pl; 2 Institute of Plant Genetics, Polish Academy of Sciences, 34 Strzeszyńska St., 60-479 Poznań, Poland; dkru@igr.poznan.pl; 3 Department of Biology and Medical Parasitology, Poznan University of Medical Sciences, 10 Fredry St., 61-701 Poznań, Poland; mderda@ump.edu.pl (M.D.); ehadas@ump.edu.pl (E.H.)

**Keywords:** alpine eryngo, in vitro shoot culture, phenolic compounds, triterpenoid saponins, phytochemical analysis, *Acanthamoeba* treatment

## Abstract

Genetically uniform shoots of *Eryngium alpinum* L. cultured in vitro were subjected to the qualitative analysis applying the UPLC-HESI-HRMS technique. In vitro cultures give the opportunity to perform the phytochemical studies on the protected species without harvesting the plant material from the natural environment. The phytochemical screening of the crude methanolic extracts of shoots, both from in vitro cultures and in vivo plants, revealed the presence of phenolic acids, coumarins, flavonoids, triterpenoid saponins, amino acids, or dipeptides. Active compounds detected are known to have medicinal importance, and for this reason, the present study represents a preliminary investigation of the extracts against pathogenic and opportunistic amoeba. Among the extracts tested, the extract of shoots from in vitro cultures exhibited remarkable amoebicidal action against trophozoites. On the second day of treatment, the extract at the concentrations of 5 mg/mL, 2.5 mg/mL, and 0.5 mg/mL showed the highest antiamoebicidal effect: the inhibition of trophozoites reached 81.14%, 66.38%, and 54.99%, respectively. To our best knowledge, the present report is the first to show the phytochemical screening and to discuss the antiamoebic activity of *Eryngium alpinum* L. shoots, both from in vitro cultures and in vivo plants.

## 1. Introduction

*Eryngium alpinum* L. is a perennial herb in the Saniculoideae subfamily of the Apiaceae family [1]. It is native the European Alps. The population of the plant is in decline. The species is protected by law: the Habitats Directive; the Convention on the Conservation of European Wildlife and Natural Habitats, the European Habitat Directive of Natura 2000, and the national red lists/books of protected species [2].

Due to the unavailability of the plant material, little research on this taxon was carried out. Only a few papers indicated the presence of phenolic acids, flavonoids and the essential oil in the organs of in vivo plants [3,4,5,6,7]. The identification of flavonoids, namely quercetin and kaempferol, in leaves of alpine eryngo was described by Crowden et al. [3]. Moreover, isoquercetin and quercitrin were detected in shoots of in vivo plants and in vitro shoot cultures [6,7]. Roots, phytochemically investigated in the study of Le Claire et al., are known to contain chlorogenic acid, R‒(+)‒rosmarinic acid and its derivative – R‒(+)‒3′‒*O*‒β‒d-glucopyranosyl rosmarinic acid [4]. Caftaric acid, chlorogenic acid, neochlorogenic acid, isochlorogenic acid, 3,4-dihydroxyphenylacetic acid, caffeic acid, and rosmarinic acid were detected in the leaves of in vivo plant and in vitro shoot cultures [6,7]. The dominant components identified in the essential oil of the aerial part of the plant were caryophyllene, bicyclogermacrene, germacrene, cariophyllene oxide, α‒bisabolol, and camphor. Furthermore, the essential oil showed a promising antiphytoviral effect, which is probably correlated with a high content of β-caryophyllene and caryophyllene oxide [5].

In vitro cultures of *E. alpinum* are a part of the collection of the protected species of the Department of Pharmaceutical Botany and Plant Biotechnology of Poznan University of Medical Sciences (PUMS). The use of in vitro cultures of alpine eryngo allows for conducting the phytochemical analyses and testing further biological activities of this species without depleting its natural sites to obtain the plant material. Since the seeds exhibit strong dormancy and a low germination rate, the generative propagation seems to be unsatisfactory to provide the raw material. In this regard, in vitro cultures of a number of the medicinal plant species offer an alternative source of uniform and renewable biomass, usually with high biosynthesis capacity for the desired compounds, with the same optical stereometry as in nature, and thus provide the valuable raw material. An important advantage of in vitro cultures is the possibility of intensifying biosynthesis and affecting the accumulation of desired metabolites in biomass, applying various biotechnological methods. It is possible to obtain biomass in a continuous large-scale production process [8].

*Acanthamoeba* is a single-celled eukaryote existing in two forms: dormant cysts and vegetative trophozoites. The infective free-living amoeba gains entry into body through eyes and ulcerated skin, which may lead to epithelial and ocular keratitis and granulomatous amoebic encephalitis. Treatment of acanthamoebosis is difficult and not always effective [9,10,11]. In humans, due to the problems in the treatment of opportunistic and pathogenic *Acanthamoeba* spp. and the lack of effective but safe drugs, the search continues for substances of plant origin that, applied as combined therapy, could contribute to decreasing the effective doses of antibiotics used [10,12,13].

The aim of the study was to obtain shoot biomass of *E. alpinum* under in vitro conditions and to conduct phytochemical analysis of the crude extracts as well as to evaluate the activity against *Acanthamoeba* sp. Shoots were developed from meristematic tissue of lateral buds. Then, they were multiplied in vitro on the artificial media by means of the axillary bud proliferation technique. The protocol of shoot multiplication allows for harvesting the high-quality and uniform raw material from alpine eryngo without decreasing the medicinal quality and quantity of bioactive compounds.

The novelty of this manuscript is the indication of the presence of coumarins, triterpenoid saponins, amino acids, dipeptides, and other compounds for the first time for this species. The manuscript aims to enrich the knowledge of phenolic compounds with unexplored phenolic acids, flavonoids, and their derivatives. The present report is the first to show complete phytochemical screening of this important taxon and discusses the antiamoebic activity of *Eryngium alpinum* L. shoots, both from in vitro cultures and in vivo plants.

## 2. Results and Discussion

### 2.1. In Vitro Shoot Culture

**Figure 1:**
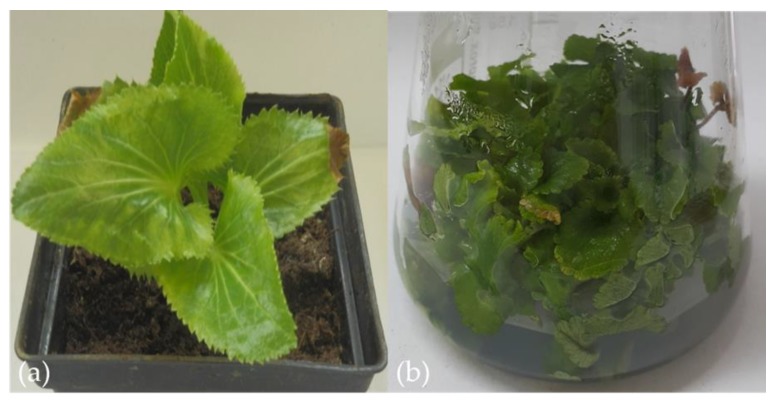
*Eryngium alpinum* L.: (**a**) shoots of in vivo plants (**b**) shoots from in vitro cultures.

*Eryngium alpinum* L. (Figure 1) was introduced into in vitro cultures and shoot cultures were established in our laboratory to study their capability of producing bioactive compounds under controlled conditions [6,7]. The biotechnological parameters of *E. alpinum* shoot multiplication under controlled conditions are presented in Table 1.

Primary explants failed to respond to MS medium without plant growth regulators, that is why this variant was withdrawn from our investigation. The hormonal investigation, regardless of the combinations and the concentration used, resulted in the response of explants (100%) and gave the largest number of new cloned shoots, with the value between 5.50 ± 0.86 and 6.79 ± 0.48. The values of the mean number of shoots calculated per one explant were not significantly different regardless of the increase in concentration of BAP and GA_3_ in the culture medium, on which shoots grew (Figure 1; Table 1).

It is worth noticing that shoots grew vigorously, did not develop roots spontaneously, and also did not show any signs of verification or callusing at base, which is important for obtaining uniform shoot biomass. This study indicated the alternative method for effective and rapid shoot multiplication of *E. alpinum*. However, the increase in the concentration of BAP did not provide the highest biotechnological parameters compared to our previous studies [6,7]. In the case of *E. planum*, the highest mean number of shoots developed via axillary buds was 15.58 ± 0.54‒17.10 ± 0.60 shoots per explant, depending on the culture media: MS + BAP 1.0 mg/L + IAA 1.0 mg/L or MS + BAP 1.0 mg/L + IAA 0.1 mg/L [14]. More shoots (13.30 ± 3.73), comparing to the control, were obtained for *E. campestre* when cultured on the same media composition as for *E. planum* [15]. The efficiency of shoot multiplication for *E. maritimum* varied between 1.2 ± 0.20 and 4.4 ± 0.24 shoots per explant on the different media variants. The highest value was observed for shoots growing on MS media supplemented with BAP 1.0 mg/L and IAA 0.1 mg/L [16].

This technique aims to obtain a large number of homogeneous plants, using only a small fragment of the donor plant, in a relatively short time. Plant multiplication via axillary bud development, as used in our experiment, provides a renewable, inexhaustible amount of the raw material, allowing for the assessment of the phytochemical profile and testing the biological activity of the extracts, which is particularly important in the case of a rare and endangered plant. In addition, it is the alternative method of clonal multiplication of a plant from a different climate zone and of a low germination rate [8].

### 2.2. The Phytochemical Analysis of Shoots from In Vitro Cultures and In Vivo Plants

**Figure 2:**
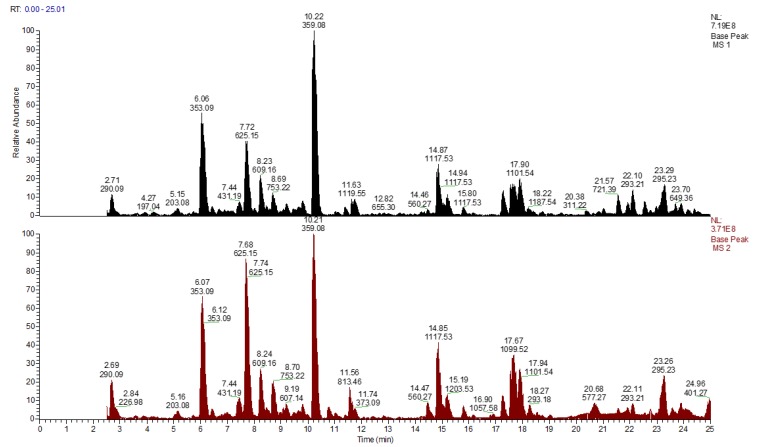
The qualitative analyses of the *Eryngium alpinum* L. samples from shoots of in vivo plants (black) and in vitro cultures (red). The base peak chromatograms in negative ion mode were obtained using the UPLC-HESI-II-HRMS system.

**Figure 3:**
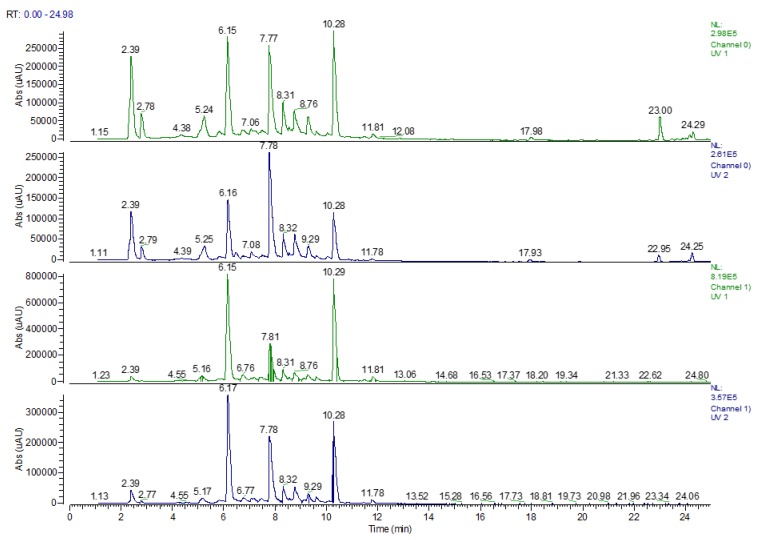
The UPLC-UV chromatograms of shoots from in vivo plants (green) and in vitro cultures (blue) of *Eryngium alpinum* L. extracts recorded at 270 nm (1,2) and 330 nm (3,4).

Shoots harvested from in vitro cultures as well as shoots from in vivo plants were subjected to the phytochemical analysis. The LC-MS base peak and the UV (270 and 330 nm) chromatograms of the *Eryngium alpinum* L. are presented in Figure 2 and Figure 3.

The retention times (RT), the observed and reference exact ion mass, the fragmentation spectra and the details are presented for the annotated compounds in Table 2. The annotation of compounds was carried out by comparing the observed and calculated exact mass for ions and the fragmentation pattern in positive and negative ion modes. Identification was complemented by applying the commercially available standards. During the analysis, 98 compounds were annotated and nine compounds were confirmed using the external standards. The main annotated compounds were phenylpropanoids, such as flavonoids (F), hydroxycinnamic acid derivates (HCA), and coumarins (C). Benzoic acid derivates (BA) and triterpenoid saponins (TT) were recognized in the samples. Other annotated groups of compounds were amino acids (AA), nucleotides (NA), carboxylic acids, some vitamins, and phytohormones.

One of the major groups of compounds were hydroxycinnamic acid derivates, which include conjugates of coumaric, caffeic and ferulic acid with the hexose (neutral losses −162.0834, C_6_H_10_O_5_) and the quinic acid (characteristic fragment *m*/*z* 191.0195, C_7_H_11_O_6_^−^). Three conjugates of caffeic acid and quinic acid were annotated in the sample namely neochlorogenic acid (5-caffeoylquinic acid, RT = 5.57 min), chlorogenic acid (3-caffeoylquinic acid, RT = 6.06), and isochlorogenic acid (5*Z*-caffeoylquinic acid, RT = 7.02 min); they were previously described by Kikowska et al. [6,7]. The pseudo-molecular ion *m*/*z* 353.08743 corresponded with the formula C_16_H_17_O_9_^−^ and gave the fragmentation pattern 191.0546, 179.0334, 161.0226, 135.0438 characteristic for caffeoylquinic acids. Compound 68 was detected in negative ion mode as a pseudo-molecular ion 515.11951, corresponded with the molecular formula C_25_H_23_O_12_^−^, and was annotated as dicaffeoylquinic acid. Compound 39 (RT = 7.14 min) was detected in negative ion mode as a coumaroylquinic acid (*m*/*z* 337.09219, C_16_H_17_O_8_^−^). The ion was observed at three different retention times (RT = 6.49, 7.76, and 8.43 min) at *m*/*z* 367.10380 (C_17_H_19_O_9_^−^), which suggested the existence of three isomeric forms of feruloylquinic acid. The conjugates of choline were detected in positive ion mode and assigned as caffeoylcholine and coumaroylcholine. The most intense peak at RT = 10.22 min was exhibited by [M − H]^−^ at *m*/*z* 359.07709 (C_18_H_15_O_8_^−^) and by a complex ion [2M − H]^−^ at *m*/*z* 719.15418. This compound was identified as rosmarinic acid by the exact mass, the fragmentation pattern and the comparison with the external standard (Sigma-Aldrich). Rosmarinic acid was previously described by Le Claire et al. [4] and Kikowska et al. [6,7]. In accordance with the literature, rosmarinic acid glucoside (521.13037, C_24_H_25_O_13_^−^) and glucuronide (535.10642, C_24_H_23_O_14_^−^) were found in negative ion mode [17]. Furthermore, 3,4-dihydroxycinnamoyl-(*Z*)-2-(3,4-dihydroxyphenyl)ethanol (313.0722, C_17_H_13_O_6_^−^) and methyl rosmarinate (373.09302, C_19_H_18_O_8_^−^) were recognized in the extracts. Coumarins such as umbeliferone, scopoletin, 7-methoxycoumarin, and dihydroxycoumarin were recognized in our sample basing on the exact mass and the fragmentation pattern and were previously described for the different *Eryngium* species and the Apiaceae family [18], however, for the first time they were recognized in *E. alpinum*. Moreover, the conjugates with glucose were tentatively identified in the samples as esculin, scopoletin-7-*O*-dihexoside, and scopolin. The pseudo-molecular ions were observed at two different retention times at *m*/*z* 223.0601 (C_11_H_11_O_5_^+^) and were tentatively assigned as isofraxidin and fraxidin.

The representative flavonoids were mostly recognized as quercetin, kaempferol, and luteolin derivates in positive and negative ion mode. The MS/MS spectra showed the typical fragmentation pattern for *O*-flavonoids with hexose (−162.0539, C_6_H_10_O_5_), rhamnose (−146.0656, C_6_H_11_O_4_), rutinose (−308.1105, C_12_H_20_O_9_), or dihexose (−324.1061, C_12_H_20_O_10_) losses in negative ion mode. Consequently, quercetin-3-*O*-rutinose, quercetin-3-*O*-galactoside, quercetin-3-*O*-glucoside, quercetin, and kaempferol were verified by means of the reference standards (Sigma-Aldrich). Other flavonoids were tentatively assigned as kaempferol-*O*-rhamnodihexoside, kaempferol-3-*O*-dihexoside, kaempferol-3-*O*-rutinoside, kaempferol-*O*-hexoside (I and II), kaempferol-3-*O*-rhamnoside, luteolin-7-*O*-rhamnohexoside, luteolin-7-*O*-hexoside, quercetin-3-*O*-dihexoside-7-*O*-rhamnoside, quercetin-3-*O*-glucoside-7-*O*-rhamnoside, and quercetin-3-*O*-dihexoside and verified with the literature data for the described *Eryngium* species [19,20]. Moreover, luteolin-*C*-hexoside, apigenin-7-*O*-rhamnohexoside, and quercetin 3-(6-*O*-acetyl)-hexoside were found in the extracts. Peak 59 showed a precursor ion at *m*/*z* 447.09238 (C_21_H_19_O_11_^−^) and was tentatively annotated as a luteolin-*C*-hexoside according to the MS/MS analysis, which corresponded to the loss of fragments−90 and −120, characterizing the break of *C*-hexoside. Compound 65 was assigned as quercetin 3-(6-*O*-acetyl)-hexoside (505.09988, C_23_H_21_O_12_^−^) whereas compound 69 was characterized as an apigenin-7-*O*-rhamnohexoside (577.16005, C_27_H_29_O_20_^−^). The product ion spectra demonstrated a fragment ion at *m*/*z* 269.0468 corresponding to apigenin aglycone. Five compounds (46, 56, 57, 63, 64), which corresponded with ions at *m*/*z*: 753.18783 (C_33_H_37_O_20_^−^), 609.14679 (C_27_H_29_O_16_^+^), 753.21992 (C_33_H_37_O_20_^−^), 607.13054 (C_27_H_28_O_16_^−^), and 739.21136 (C_33_H_39_O_19_^+^), were recognized as unknown flavonoids. The MS/MS spectrum of compounds 55, 65, 66 and 72 showed a major fragment at *m*/*z* 301.03711 (C_15_H_9_O_7_^−^) in negative ion mode, which could be quercetin aglycone. Furthermore, a fragment at *m*/*z* 285.03955 (C_15_H_9_O_6_^−^) was found in the MS/MS spectrum of compound 64 and corresponded with the tetrahydroxyflavone moiety.

Several triterpenoid saponins were found in the samples. Similar to Ożarowski et al. [17], we observed precursors and the fragmentation pattern as in the related species, *E. planum*. The pseudo-molecular ion at *m*/*z* 471.34903 corresponded with the molecular formula C_30_H_47_O_4_^−^ and was recognized as a major sapogenin in the extract. Twelve precursors were tentatively assigned as triterpenoid saponins. The pseudo-molecular ions 1119.55419 (C_54_H_87_O_24_^−^) and 1099.52958 (C_54_H_83_O_23_^−^) could be annotated as eryngioside C and eryngioside J according to the exact mass and fragmentation [21]. Moreover, ions 969.51023 (C_49_H_77_O_19_^+^) and 911.50453 (C_47_H_75_O_17_^+^) could be putatively assigned as 3-*O*–β-d-glucopyranosyl-(1→2)-β-d-glucuronopyranosyl-22-*O*-angeloyl-R1-barrigenol and 3-*O*-β-d-glucopyranosyl-(1→2)-β-d-glucuronopyranosyl-22-*O*-angeloyl-A1-barrigenol – saponins observed in *E. planum* and *E. maritimum* [22]. This is the first report on triterpenoid saponins detection in *E. alpinum*. However, further works are required for identification of the saponin structures in this species.

In line with the literature, hydroxybenzoates were found in the extracts of *Eryngium* [20]. Glucosyringic acid (359.09950, C_15_H_19_O_10_^−^), trimethoxybenzoic acid (213.07613, C_10_H_13_O_5_^+^), dihydroxybenzoic acid hexoside (315.0725, C_13_H_15_O_9_^−^), vanillic acid hexoside (329.08841, C_14_H_17_O_9_^−^), vanillic acid (167.0350, C_8_H_7_O_4_^−^), hydroxyphenyllacetic acid (181.05003, C_9_H_9_O_4_^−^), dimethoxybenzaldehyde (167.07097, C_9_H_11_O_3_^+^), and glucovanillin (313.09305, C_14_H_17_O_8_^−^) were tentatively recognized in the sample. Also, citric acid was identified as a major carboxylic acid. Twelve amino acids and dipeptides were observed in positive and negative ion mode. Three nucleotides were recognized as uridine, adenosine and guanosine. The pseudo-molecular ions at *m*/*z* 182.04490 (C_8_H_8_NO_4_^−^) and 218.10310 (C_9_H_16_NO_5_^−^) were annotated as 4-pyridoxic acid and pantothenic acid, the major vitamins in the extracts. Some of phytohormones such as 5-hydroxy-3-indoleacetic acid, gibberellic acid, 12-hydroxyjasmonic acid, jasmonic acid, and OPDA were putatively identified by means of the exact mass and the fragmentation pattern.

**Figure 4:**
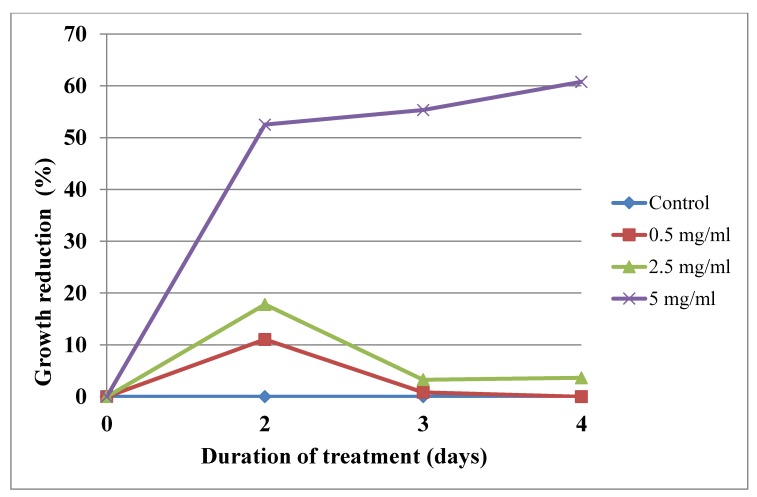
The effect of the extract from shoots of in vivo plant *Eryngium alpinum* L. [0.5 mg/mL, 2.5 mg/mL, 5 mg/mL] on inhibition of *Acanthamoeba* trophozoites proliferation in the culture medium.

**Figure 5:**
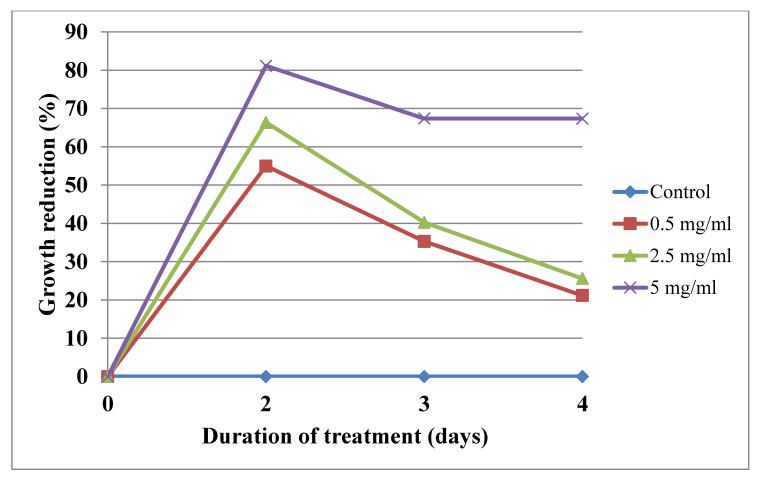
The effect of the extract from in vitro shoot cultures of *Eryngium alpinum* L. [0.5 mg/mL, 2.5 mg/mL, 5 mg/mL] on inhibition of *Acanthamoeba* trophozoites proliferation in the culture medium.

The results of the study indicated that the extracts obtained from *E. alpinum* shoots, both from in vitro cultures in vivo plantlets, inhibited growth of *Acanthamoeba* sp. trophozoites to varying degrees (Table 3, Table 4 and Table 5; Figure 4 and Figure 5).

The dependence of the effect on the extract concentration and treatment time was noted. The strongest effect was observed for leaves from in vitro shoot culture. The extract showed the highest antiamoebicidal effect already on the second day of treatment: indicated inhibition of trophozoites was 81.14%, 66.38%, and 54.99% at the concentrations of 5 mg/mL, 2.5 mg/mL, and 0.5 mg/mL, respectively (Table 4, Figure 4). The extract from shoots of in vivo plants at a dose of 0.5 and 2.5 mg/mL weakly inhibited the development of trophozoites (Table 3, Figure 3). The best IC_50_ index was calculated for leaves from the shoot culture extract. On the second day of treatment, the IC_50_ value was 0.35 mg/mL (Table 5).

Due to the problems in the treatment of opportunistic *Acanthamoeba* spp. and the lack of effective but safe drugs, the search continues for substances of plant origin that, applied as combined therapy, could contribute to decreasing the effective doses of antibiotics used [10,12].

In the literature on the subject, more scientific information on the plant extracts with the amoebicidal or amoebistatic activity against pathogenic strains of Acanthamoeba spp. can be found regarding the extracts from leaves of *Origanum* spp., *Salvia* spp., *Melia azedarach, Ricinus communis*, *Pastinaca armenea, Inula oculuscristi*; aerial parts of *Croton* spp., *Pterocaulon polystachyum*, flowers, roots and leaves of *Rubus chamaemourus*, *Pueraria lobata, Solidago* spp., flowers, roots, leaves and bark of *Ipomoea* sp., *Kaempferia galanga, Cananga odorata*, leaves and calluses of *Passiflora* spp., leaves and roots of *Eryngium planum* [9,11,23,24,25,26,27].

It was shown in our studies that the extract of leaves from in vitro shoot culture of E. alpinum at a dose of 0.5 mg/mL was effective in inhibiting trophozoites, which can be interpreted as favourable compared to the amoebicidal effect of the plant extracts such as *Allium sativum* at 3.9 mg/mL [28], *Salvia staminea* at 16 mg/mL [29], *Peucedanum caucasicum*, *P*. *palimbioides*, *P*. *chryseum*, *P*. *longibracteolatum* [30], *Origanum syriacum*, O. *laevigatum* [31], *Buddleia cordata* at 32 mg/mL [32], and *Trigonella foenum-graecum* at 400 mg/mL [33].

The flavonoid-saponin fraction of the ethanolic extract from leaves of *Eryngium planum* L., at the concentration of 1 mg/mL, with the similar phytochemical pattern to *E. alpinum*, showed the amoebistatic effect—76% inhibition of amoebae growth on the third day of treatment. However, the flavonoid fraction from leaves at the concentration of 5 mg/mL revealed the 56.1% inhibitory effect and the phenolic acid fraction at the concentration of 2 mg/mL showed 36.8% inhibition. The authors concluded that the activity may be correlated with the saponin actions, which may be associated with the integration between those compounds and the cell wall of *Acanthamoeba* [27]. As stated by Mahboob et al. [34], the acanthamoebicidal effect of *Lonicera japonica* flower, which evoked a significant reduction of trophozoite viability, depends mostly on the major compound form the extract, that is chlorogenic acid. According to Bittner Fialová, rosmarinic acid and its derivates appeared to be promising anti-*Acanthamoeba* agents with the EC_50_ values between 5.6 ± 0.3 mM and 19.1 ± 0.4 mM [35]. The biological study of the *Passiflora* spp. extracts from leaves and callus biomass revealed that all the samples showed amoebostatic and amoebicidal properties at the concentrations from 4 to 12 mg/mL. The authors tried to find a correlation between the studied activity and the presence of phenolic compounds, with particular emphasis on flavonoids [26]. Moreover, it is noteworthy that quercetin exhibited potent antiamoebic activities against *Acanthamoeba* [36]. These findings were accordingly similar to the results of the study performed on fractions of the ethanol extracts prepared from *Frankenia thymifolia*. The fractions showed moderate activity against *Acanthamoeba castellanii*, which may be associated with the presence of quercetin and its derivatives [37]. As it was shown in our study, *E. alpinum* shoots, in addition to the presence of phenolic acids and flavonoids, are characterized by a broad spectrum of coumarins. And as it results from numerous studies, phenolic compounds in the extracts of the species such as *Allium sativum, Solidago virgaurea*, *Teucrium chamaedrys* or *Peucedanum* spp. are responsible for the amoebicidal effect [11].

To our best knowledge, the present report is the first one that discusses the phytochemical screening and discusses the antiamoebic activity of *Eryngium alpinum* L. shoots from in vitro cultures and in vivo plants of this endangered species.

## 3. Materials and Methods

### 3.1. The Plant Material Origin

The fragments of the cuttings of *Eryngium alpinum* L. obtained from the Botanical Garden of Adam Mickiewicz University in Poznań in 2017 (52°25′13.1′′N 16°52′44.9′′E) were used for the initiation of in vitro cultures. The voucher specimens were deposited at the Department of Pharmaceutical Botany and Plant Biotechnology of PUMS under the following number: H‒AP‒2017‒102.

### 3.2. Establishment of In Vitro Cultures

Young shoots with lateral buds were harvested. The collected explants were disinfected and transferred into basal MS medium [38] with plant growth regulators (PGRs), namely cytokinin BAP (6-benzylaminopurine), auxin IAA (indole-3-acetic acid), and gibberellin GA_3_ (gibberellic acid) at the concentration of 1.0 mg/l (Table 1), 0.76% agar and pH set to 5.8 before autoclaving at 121 °C, 105 kPa for 20 min. All PGRs and agar originated from Sigma-Aldrich (Saint Louis, MO, USA). The cultures were placed in a growth chamber under controlled conditions, i.e., 21 °C with a 16 h light/8 h dark photoperiod, 55 µmol/m^2^s light, and subcultured every five weeks. Multiplication of shoots was repeated three times for each hormonal treatment using at least 10 explants (2–3 per flask).

### 3.3. Detection of Metabolites in the Extracts Using UPLC-HESI-II-HRMS

In order to conduct the phytochemical analysis, the exact amounts of fresh biomass from basal leaves of the intact plants as well as shoots from the in vitro cultures were dried at 40 °C for 24 h to a constant weight. The dried samples were extracted with 70% (*v*/*v*) EtOH (25 mg DW to 2.0 mL) in safe-lock tubes (Eppendorf, Hamburg, Germany). The samples were shaken at 3000 rpm for 20 min (IKA MS 3 Basic Vortex Mixer, Staufen, Germany) and centrifuged at 12,000 rpm, at 4° C for 15 min (Allegra 21 centrifuge, Beckman Coulter, Brea, CA, USA). Supernatants were filtered through a 0.22 μm PTFE syringe filter (Φ 13 mm, Kinesis Ltd, St. Neots, U.K.). Aquity UPLC (Waters, Milford, MA, USA) with a high resolution Orbitrap mass spectrometer (Thermo Fischer, Bremen, Germany) were applied to the phytochemical analysis of the ethanolic extracts. BEH C13 column (1.7 µm, 2.1 × 150 mm, Waters) was used for separation of the samples (3 µL, partial loop mode) at 45 °C column temperature and 300 µL/min flow rate. 0.1% of formic acid in water (solvent A, MiliQ system, Merck, Darmstadt, Germany) and acetonitrile (solvent B, LC/MS grade, Merck) were used in gradient: initial—5% B, 20 min—75% B, 22 min—98% B, and isocratic 98% B for 24 min. The PDA detector scanned in the range 220–400 nm at frequency 20 spectra/s.

The Orbitrap mass spectrometer equipped with the heated electrospray ion source II (HESI-II) operated in negative and positive ion mode. HESI II settings were: capillary voltage—2.5 kV (negative) and 3.5 kV (positive), sheath gas flow—35, auxiliary gas flow—10, sweep gas flow—3 arbitrary units, ion transfer tube temperature—400 °C, auxiliary gas heater temperature—350 °C, and S-lens RF level 50. The full-MS spectra were recorded at mass resolution of 70,000 in the range 150–2000 *m*/*z* and 200 ms maximum inject time. The data dependent MS2 spectra were recorded at resolution of 17,500. The data files were processed using Xcalibur Qual Browser (Thermo Fischer) and MSDIAL 3.9 software [39].

### 3.4. Acanthamoebic Activity Examination

In order to conduct the biological analysis, the exact amounts of fresh biomass from basal leaves of the intact plants as well as shoots from the in vitro cultures were dried at 40 °C for 24 h to a constant weight. Dried shoots from in vivo plants and in vitro cultures were extracted three times with EtOH 70% (*v*/*v*) at 95 °C. The extracts were concentrated under reduced pressure and used for the evaluation of the antiamoebic studies. The extract samples were weighed and then dissolved in 40 mL of DMSO (dimethyl sulfoxide). Distilled water was added to the solution to obtain the appropriate concentration. Then 200 mL of the appropriately diluted solution was added to 2 mL of trophozoites cultures to obtain the expected final concentrations (0.5, 2.5 and 5 mg/mL). In this study the strain of *Acanthamoeba*, isolated from the environmental sample, was used. This *Acanthamoeba* sp. strain was deposited in GenBank (NCBI) under the accession number KY203908. The pathogenicity of this strain was tested on laboratory animals. The research showed that this strain of amoebae is pathogenic for mice. The amoebae were axenically cultured on the liquid medium containing 2% Bacto-Casitone. Parasitological examination of the extracts was performed according to Derda et al. [9]. The study investigated the activity of the ethanol extracts from in vitro shoot cultures and in vivo plants. The increase in the number of parasites in culture was studied. Thoma hemocytometry chamber was used for cell counting. The amoebae were counted three times at 24 h intervals. The control consisted of cultured trophozoites without any extracts. The relationship between the extract concentration and the time of treatment for amoebae cultures was investigated.

### 3.5. The Statistical Analysis

The mean number of *E. alpinum* shoots and their length as well as the standard error were calculated in each hormonal variant of the culture medium. The data from biotechnological experiments were analyzed using a one-way analysis of variance (ANOVA) and the statistical significance was determined using Duncan’s POST-HOC test (*p*-value < 0.05). All the analyses were conducted employing STATISTICA v. 13 (StatSoft, Inc. 2015). The mean number of amoebae and standard deviation were calculated in each measurement group. The statistical analysis was determined employing the Mann-Whitney and ANOVA tests. Statistical significance was defined as *p* < 0.05.

## 4. Conclusions

In vitro shoot culture of *Eryngium alpinum* L. can be considered a valuable alternative source of biomass that is rich in desired secondary metabolites such as phenolic acids, coumarins, flavonoids, and triterpenoid saponins. This is especially important for protected species, the collection of which from the natural environment is impossible. The results suggest that the extracts from *E. alpinum* may be promising natural products for *Acanthamoeba* treatment. Further studies are necessary to clarify which bioactive compounds are responsible for the observed activity.

## Figures and Tables

**Table 1:** The effect of selected plant growth regulators—BAP (6-benzylaminopurine), IAA (indole-3-acetic acid) and GA_3_ (gibberellic acid) present in MS media on shoot multiplication ratio and length of multiplied shoots of *Eryngium alpinum* L. after 40 days of culture.

No.	Cytokinin [mg/L]	Gibberellin [mg/L]	Auxin [mg/L]	Mean No. of Shoots ± SE	Mean Length of Shoots [cm] ± SE
1.	BAP 2.0	GA_3_ 1.0	-	6.56 ± 0.35 ^ns^	2.85 ± 0.06 ^a,b^
2.	BAP 2.0	GA_3_ 1.0	IAA 1.0	6.79 ±0.48	2.57 ± 0.80 ^b^
3.	BAP 1.0	GA_3_ 1.0	IAA 1.0	6.33 ± 1.21	2.08 ± 0.21 ^c^
4.	BAP 1.0	GA_3_ 0.5	IAA 1.0	5.50 ± 1.04	3.03 ± 0.20 ^a^
5.	BAP 1.0	GA_3_ 0.5	IAA 1.0	5.50 ± 0.86	3.01 ± 0.15 ^a,b^

Mean values within a column with the same letter are not significantly different at *p* < 0.05. (Duncan’s Multiple Range Test).

**Table 2:** The annotated compounds in the shoot extracts of *Eryngium alpinum* L. from in vitro culture and in vivo plant. Metabolites detected by UPLC-HESI-II-HRMS. The compounds were characterized by the exact mass and the fragmentation pattern in negative and positive ion mode.

No	RT [min]	Ion mode	Observed *m*/*z*	Reference *m*/*z*	Delta (ppm)	Molecular Formula	Fragmentation	Name	MSI Level ^a^	Class ^b^	CID^c^	Ref.
1	2.58	[M + H]^+^	175.11955	175.11896	3.4	C_6_H_14_N_4_O_2_	60.0564, 70.0660, 116.0717, 160.0981, 158.0928, 158.0921,	Arginine	3	AA	6322	
2	2.7	[M − H]^−^	191.0191	191.01973	−3.3	C_6_H_8_O_7_	57.0329, 111.0069, 129.0175, 191.0193,	Citric acid	1 ^s^	CA	311	
3	2.7	[M − H]^−^	243.06236	243.06226	0.4	C_9_H_12_N_2_O_6_	82.0280, 110.0228, 122.0231, 140.0340, 152.0331, 200.0553.	Uridine	2	N	6029	
4	2.7	[M − H]^−^	180.0657	180.06662	−5.1	C_9_H_11_NO_3_	72.0073, 93.0335, 119.0484, 163.0383, 180.0649,	Tyrosine	1 ^s^	AA	6057	3
	2.7	[M + H]^+^	182.0817	182.08118	2.9	C_9_H_11_NO_3_	119.0493, 123.0442, 136.0758, 147.0441, 165.0553, 182.0824,					
5	2.7	[M + H]^+^	268.10495	268.10403	3.4	C_10_H_13_N_5_O_4_	57.0346, 136.0624, 268.1039,	Adenosine	2	N	60961	
6	2.73	[M − H]^−^	282.08435	282.08438	−0.1	C_10_H_13_N_5_O_5_	133.0141, 150.0403,	Guanosine	2	N	135398635	
7	2.93	[M + H]^+^	276.14529	276.1445	2.9	C_12_H_23_NO_7_	86.0970, 132.1028, 212.1273, 230.1401, 258.1352, 276.1455	*N*-Fructosyl isoleucine	3	AA	137530247	
8	3	[M − H]^−^	182.0449	182.04588	−5.4	C_8_H_9_NO_4_	108.0445, 120.0439, 138.0539, 182.0458	4-Pyridoxic acid	2	O	6723	
9	3.65	[M + H]^+^	192.06627	192.06552	3.9	C_10_H_9_NO_3_	146.0598, 192.1035	5-Hydroxy-3-indoleacetic acid	3	PH	1826	
10	3.76	[M + H]^+^	175.11923	175.11896	1.5	C_6_H_14_N_4_O_2_	60.0564, 116.0717, 160.0981, 158.0928, 158.0921	Arginine	3	AA	6322	
11	3.77	[M + H]^+^	209.09282	209.09207	3.6	C_10_H_12_N_2_O_3_	74.0249, 120.0449, 136.0759, 146.0599, 163.0859, 174.0547, 192.0658,	Kynurenine	2	AA	846	
12	3.92	[M − H]^−^	359.0995	359.0977	5	C_15_H_20_O_10_	-	Glucosyringic acid	3	BA	-	4
13	4.01	[M − H]^−^	338.08862	338.08789	2.2	C_15_H_17_NO_8_	132.0443, 176.0334	Indole + 1*O*, 1carboxy, *O*-Hex;	3	AA	-	
14	4.17	[M + H]^+^	213.07613	213.07574	1.8	C_10_H_12_O_5_	149.0606, 195.0643	Trimethoxybenzoic acid	3	BA	-	
15	4.21	[M − H]^−^	315.0725	315.071	4.8	C_13_H_16_O_9_	108.0199, 152.0102, 315.0696	Dihydroxybenzoic acid hexoside	3	BA	-	4
16	4.26	[M − H]^−^	167.035	167.03499	0.1	C_8_H_8_O_4_	108.0200, 123.0434, 152.0102, 167.0343	Vanillic acid	2	BA	8468	
17	4.26	[M + H]^+^	179.03462	179.03389	4.1	C_9_H_6_O_4_	105.6870, 107.0502, 123.0445, 133.0281, 151.0394, 179.1061	Dihydroxycoumarin	2	C	-	
18	4.26	[M − H]^−^	329.08838	329.08841	−0.1	C_14_H_18_O_9_	108.0200, 123.0434, 152.0102, 167.0343	Vanillic acid hexoside	3	BA	-	
19	4.39	[M − H]^−^	218.1031	218.10339	−1.3	C_9_H_17_NO_5_	71.0121, 88.0389, 99.0432, 116.0707, 140.2067	Pantothenic acid	3	O	6613	
20	4.95	[M]^+^	266.1389	266.13812	2.9	C_14_H_20_NO_4_	95.0861, 163.0382, 207.0648	Caffeoylcholine	2	HC	6440794	
21	5.16	[M − H]^−^	203.08162	203.0826	−4.8	C_11_H_12_N_2_O_2_	116.0494, 142.0644, 159.0909, 186.0549,	Tryptophan	1 ^s^	AA	6305	3
	5.16	[M + H]^+^	205.09726	205.09715	0.5	C_11_H_12_N_2_O_2_	118.0661,132.0812, 146.0598, 159.0912, 170.0601, 188.0714,					
22	5.18	[M]^+^	250.1441	250.14322	3.5	C_14_H_20_NO_3_	-	Coumaroylcholine	2	HC	6440550	
23	5.3	[M − H]^−^	181.05003	181.05063	−3.3	C_9_H_10_O_4_	72.9914, 119.0483, 135.0436, 163.0382, 181.0498	Hydroxyphenyllactic acid	3	BA	-	
24	5.42	[M − H]^−^	339.07239	339.07214	0.7	C_15_H_16_O_9_	177.0192,	Esculin	2	C	5281417	
25	5.57	[M − H]^−^	353.08743	353.0878	−1	C_16_H_18_O_9_	161.0231, 173.0437, 179.0333, 191.0195,	Neochlorogenic acid	1 ^s^	HC	5280633	1,3
26	5.58	[M − H]^−^	355.0672	355.06561	4.5	C_15_H_16_O_10_	135.0794, 147.0285, 163.0382, 179.0697, 191.0199, 209.0293	Coumaroyl + C_6_H_9_O_8_	3	HC	-	
27	5.7	[M + H]^+^	167.07097	167.07027	4.2	C_9_H_10_O_3_	111.0448, 139.0757, 149.0232, 167.0708	Dimethoxybenzaldehyde	3	BA	-	
28	5.71	[M + H]^+^	261.14514	261.1445	2.5	C_11_H_20_N_2_O_5_	84.0451, 86.0605, 132.1017, 198.1126, 244.1189, 261.1274	Glutamylleucine	2	AA	9856500	
29	5.73	[M + H]^+^	517.15778	517.15574	4	C_22_H_28_O_14_	193.0489, 178.0266, 165.0556, 133.0285	Scopoletin 7-*O*-dihexoside	2	C	-	
30	5.91	[M − H]^−^	341.08795	341.08726	2	C_15_H_18_O_9_	119.0336, 161.0236, 179.0339,	Caffeic acid glucoside	2	HC	5281761	3
31	6.06	[M + H]^+^	355.1022	355.10236	−0.5	C_16_H_18_O_9_	163.0385,	Chlorogenic acid	1 ^s^	HC	1794427	3
	6.06	[M − H]^−^	353.08755	353.0878	−0.7	C_16_H_18_O_9_	135.0437, 161.0226, 179.0334, 191.0546					
32	6.17	[M + H]^+^	295.12949	295.12949	0	C_14_H_18_N_2_O_5_	120.0811, 166.0859, 186.0907, 232.0963	Glutamylphenylalanine	2	AA	111299	
33	6.3	[M − H]^−^ ]^−^	399.09326	399.09299	0.7	C_16_H_18_O_9_	135.0437, 176.0116, 191.0346, 221.0073	Scopolin	2	C	439514	
34	6.49	[M − H]^−^	367.1038	367.10199	4.9	C_17_H_20_O_9_	134.0357, 149.0238, 163.0483, 191.0562, 193.0493	Feruloylquinic acid	3	HC	10177048	3,4
35	6.5	[M − H]^−^	355.0665	355.06509	4	C_15_H_16_O_10_	147.0282, 191.0194, 209.0293	Coumaroyl + C_6_H_9_O_8_	3	HC	-	
36	6.77	[M + H]^+^	223.06041	223.0601	1.4	C_11_H_10_O_5_	149.0244, 162.0302, 177.0907, 190.0266, 207.0280,	Isofraxidin	2	C	5318565	
37	7.00	[M + H]^+^	773.21649	773.2135	3.9	C_33_H_40_O_21_	132.1144, 228.9691, 303.0504	Quercetin-3-*O*-dihexoside-7-*O*-rhamnoside	3	F	57393076	
		[M − H]^−^	771.20292	771.19839	5.9		151.0028, 178.9972, 300.0273, 446.0863, 625.1580					
38	7.02	[M − H]^−^	353.08746	353.08621	3.5	C_16_H_18_O_9_	135.0437, 161.0226, 179.0334, 191.0546(100%)	Caffeoylquinic acid (Isochlorogenic acid)	2	HC	5315832	1
		[M + H]^+^	355.10339	355.10236	2.9		137.0612, 163.0386, 201.0543					
39	7.14	[M − H]^−^	337.09293	337.09219	2.2	C_16_H_18_O_8_	163.0483, 191.0562	Coumaroylquinic acid	2	HC	6441280	3
40	7.41	[M + H]^+^	611.16309	611.16121	3.1	C_27_H_30_O_16_	303.0502	Quercetin-3-*O*-hexoside-7-*O*-rhamnoside	2	F	25080064	2
		[M − H]^−^	609.15906	609.15612	4.8		299.0215, 301.0368, 447.0932, 463.0879					
41	7.49	[M − H]^−^	345.13425	345.13437	−0.3	C_19_H_22_O_6_	143.0849, 221.1323, 239.1435, 273.1489	Gibberellic acid	3	O	6466	
42	7.51	[M − H]^−^	755.2341	755.23986	−7.6	C_33_H_40_O_20_	176.8649, 227.0346, 255.0293, 284.0326, 285.0404, 609.1585	Kaempferol-*O*-rhamnodihexoside	3	F	-	
		[M + H]^+^	757.2229	757.21913	5.0		287.0552					
43	7.71	[M − H]^−^	625.14482	625.14105	6	C_27_H_30_O_17_	151.0014, 178.9977, 255.0304, 271.0252, 300.0289, 301.0323	Quercetin-3-*O*-dihexoside	2	F	14185727	4
	7.71	[M + H]^+^	627.15741	627.15558	2.9	C_27_H_30_O_17_	159.8611, 281.4450, 303.0503					
44	7.74	[M + H]^+^	449.10812	449.10785	0.6	C_21_H_20_O_11_	130.1475, 287.0541, 299.0568, 329.0639, 353.0654	Luteolin-*C*-hexoside	3	F	-	
	7.74	[M − H]^−^	447.09238	447.09329	−2	C_21_H_20_O_11_	285.0380, 297.0394, 327.0507, 357.0601					
45	7.76	[M − H]^−^	367.1038	367.10291	2.4	C_17_H_20_O_9_	134.0357, 173.0386, 191.0546	Feruloylquinic acid	3	HC	10177048	3,4
		[M + H]^+^	369.11871	369.11801	1.9		117.0337, 145.0289, 149.0607, 163.0385, 177.0550, 195.0642					
46	7.86	[M + H]^+^	755.20820	755.20660	4.2	C_33_H_38_O_20_	127.0398, 145.0505, 303.0504	Unknown flavonoid	3	F	-	
		[M − H]^−^ ]^−^	753.18783	753.19064	−3.7		301.0349, 446.0853, 463.0864					
47	8.03	[M + H]^+^	227.12856	227.12779	3.4	C_12_H_18_O_4_	, 131.0861, 149.0969, 167.1077, 191.1070, 209.1177, 227.1267	12-Hydroxyjasmonic acid	2	O	5497122	
48	8.17	[M − H]^−^	172.09709	172.09792	−4.8	C_8_H_15_NO_3_	130.0862, 172.0976	*N*-Acetylleucine	2	AA	70912	
49	8.25	[M − H]^−^	609.14894	609.14612	4.6	C_27_H_30_O_16_	151.0018, 163.0026, 178.9970, 227.0341, 255.0299, 284.0318, 285.0379, 609.1639	Kaempferol-3-*O*-dihexoside	2	F	5282155	4
		[M + H]^+^	611.16367	611.16064	5		127.0398, 287.0541					
50	8.4	[M − H]^−^	192.066	192.06662	−3.2	C_10_H_11_NO_3_	-	Phenylacetylglycine	2	AA	68144	
51	8.43	[M − H]^−^ ]^−^	367.1038	367.10199	4.9	C_17_H_20_O_9_	191.0546	Feruloylquinic acid	3	HC	10177048	
52	8.47	[M − H]^−^	609.14887	609.14612	4.5	C_27_H_30_O_16_	133.8210, 151.0018, 177.9567, 255.0308, 271.0236, 285.0425, 300.0290, 301.0323, 609.1639	Quercetin-3-*O*-rutinoside	1 ^s^	F	5280805	1
		[M + H]^+^	611.16321	611.16122	3.3		129.0572, 287.0541, 303.0504					
53	8.67	[M − H]^−^	463.08749	463.0882	−1.5	C_21_H_20_O_12_	151.0036, 255.0304, 271.0255, 287.2002, 300.0289, 301.0315	Quercetin-3-*O*-galactoside	1 ^s^	F	5281643	
		[M + H]^+^	465.10483	465.10275	4.5		142.7517, 257.0451, 285.0396, 303.0503					
54	8.67	[M − H]^−^	593.15129	593.15118	0.2	C_27_H_30_O_15_	227.0337, 255.0302, 272.9905, 284.0320, 285.0383, 593.1517	Kaempferol-3-*O*-rutinoside	2	F	5318767	2
		[M + H]^+^	595.16882	595.16577	5.1		85.0296, 164.3077, 253.8810, 287.0542					
55	8.67	[M − H]^−^	313.09305	313.09235	2.3	C_14_H_18_O_8_	121.0280	Glucovanillin	3	BA	6452133	
56	8.68	[M + H]^+^	609.14679	609.14557	2.0	C_27_H_28_O_16_	303.0501	Unknown	3	F	-	
57	8.7	[M − H]^−^	753.21992	753.22421	−5.7	C_33_H_38_O_20_	151.0018, 255.0304, 271.0245, 300.0290, 301.0324 609.1639	Unknown flavonoids	3	F	11498684	
		[M + H]^+^	755.20740	755.20347	5.2		179.3386, 303.0505					
58	8.77	[M − H]^−^	463.08752	463.0882	−1.5	C_21_H_20_O_12_	151.0020, 255.0296, 271.0252, 300.0288, 301.0371, 463.0862	Quercetin-3-*O*-glucoside	1^s^	F	5280804	1,4
		[M + H]^+^	465.10287	465.10275	0.3		257.0451, 275.6729, 285.0396, 303.0504, 465.1721					
59	8.96	[M − H]^−^	447.09442	447.09329	2.5	C_21_H_20_O_11_	167.4627, 279.2310, 284.0321, 285.0379, 447.0933	Luteolin 7-*O*-glucoside	3	F	5280637	2
60	9.01	[M − H]^−^	245.09334	245.09317	0.7	C_13_H_14_N_2_O_3_	-	*N*-Acetyltryptophan	3	AA	2002	
61	9.02	[M − H]^−^	521.13037	521.12952	1.6	C_24_H_26_O_13_	135.0531, 161.0233, 179.0336, 197.0438	Rosmarinic acid glucoside	2	HC	132604855	3
62	9.12	[M + H]^+^	223.06041	223.0601	1.4	C_11_H_10_O_5_	121.0291, 149.0244, 162.0302, 177.0907, 190.0266, 207.0280	Fraxidin	2	C	3083616	
63	9.19	[M + H]^+^	609.14673	609.14556	1.9	C_27_H_28_O_16_	303.0503	Unknown flavonoids	3	F	-	
		[M − H]^−^	607.13054	607.12991	−1.0		151.0025, 178.9976, 255.0293, 271.0252, 300.0273, 463.0867					
64	9.19	[M + H]^+^	739.21136	739.20856	3.8	C_33_H_38_O_19_	287.0552	Unknown flavonoids	3	F	-	
		[M − H]^−^	737.20084	737.19291	9.8		151.0023, 255.0289, 284.0325, 285.0395					
65	9.23	[M − H]^−^	505.09988	505.09875	2.2	C_23_H_22_O_13_	151.0018, 271.0246, 300.0292, 301.0322	Quercetin 3-(6-*O*-acetyl)-hexoside	2	F	10006384	
66	9.25	[M − H]^−^	593.15141	593.15118	0.4	C_27_H_30_O_15_	133.0971, 151.0018, 255.0304, 284.0320, 285.0382	Luteolin-7-*O*-rhamnohexoside	2	F	5318767	2
		[M + H]^+^	595.16858	595.16630	2.9		287.0552					
67	9.29	[M − H]^−^	449.10779	449.10730	1.1	C_21_H_20_O_11_	287.0544	Kaempferol-*O*-hexoside I	2	F	5282149	2
			447.09445	447.09329	2.6		150.1186, 196.2700, 227.0370, 255.0304, 284.0321, 285.0380					
68	9.55	[M − H]^−^	515.12018	515.11951	1.3	C_25_H_24_O_12_	135.0437, 161.0230, 179.0334, 191.0546, 353.0867	Dicaffeoylquinic acid	3	HC	13604687	3
		[M + H]^+^	517.13562	517.13403	3.1		135.0442, 145.0291, 163.0386					
69	9.56	[M − H]^−^	577.16005	577.15625	6.6	C_27_H_30_O_14_	269.0468	Apigenin-7-*O*-rhamnohexoside	2	F	5282150	
		[M + H]^+^	579.17301	579.17084	3.7		85.0294, 200.8167, 271.0601					
70	9.67	[M − H]^−^	447.09457	447.09329	2.9	C_21_H_20_O_11_	227.0337, 249.0604, 255.0309, 279.2349, 284.0317, 285.0379	Kaempferol-*O*-hexoside II	2	F	5282102	2
		[M − H]^−^	447.0943	447.09329	2.3		279.2305, 285.0385					
71	10.03	[M − H]^−^	535.10642	535.10879	−4.4	C_24_H_24_O_14_	135.0436, 179.0335, 197.0448	Rosmarinic acid, glucuronide	2	HC	-	3
72	10.07	[M − H]^−^	351.0724	351.07214	0.7	C_16_H_16_O_9_	-	4-Methylumbelliferyl glucuronide	3	C	91553	
73	10.13	[M − H]^−^	191.03415	191.0341	0.3	C_10_H_8_O_4_	-	Coumarin base + 1*O*, 1MeO	3	C	-	
74	10.22	[M − H]^−^	359.07709	359.07724	−0.4	C_18_H_16_O_8_	72.9915, 135.0437, 161.0231, 179.0334, 197.0434	Rosmarinic acid	1 ^s^	HC	5281792	3
75	10.23	[M − H]^−^	313.0722	313.07122	3.1	C_17_H_14_O_6_	109.0279, 123.0430, 133.0286, 151.0387, 161.0231	3,4-Dihydroxycinnamoyl-(*Z*)-2-(3,4-dihydroxyphenyl)ethenol	2	HC	14353342	
76	10.61	[M − H]^−^	431.09967	431.09836	3	C_21_H_20_O_10_	152.9944, 227.0337, 255.0304, 285.0388	Kaempferol-3-*O*-rhamnoside	2	F	5316673	2
77	10.71	[M − H]^−^	193.04984	193.05063	−4.1	C_10_H_10_O_4_	121.0280, 133.0775, 148.8996, 161.02284, 177.0188,	Ferulic acid	2	HC	445858	
	10.75	[M + H]^+^	195.06569	195.06519	2.6	C_10_H_10_O_4_	135.0446,145.0289, 149.0605, 163.0385, 177.0550					
78	11.08	[M − H]^−^	191.03412	191.03499	−4.6	C_10_H_8_O_4_	147.0439, 149.0238, 175.0114, 191.0345	Scopoletin	2	C	5280460	2
	11.08	[M + H]^+^	193.05025	193.04953	3.7	C_10_H_8_O_4_	-					
79	11.4	[M − H]^−^	1251.60254	1251.60100	1.2	C_59_H_96_O_28_	589.4164, 633.4128, 733.4748, 751.4918, 865.5422, 883.5467 927.5394, 957.5598, 1045.6177, 1089.5382, 1251.6044	Triterpenoid saponin	3	TT	-	
80	11.63	[M − H]^−^	1119.55419	1119.55874	−4.1	C_54_H_88_O_24_	589.4158, 633.4137, 733.4717, 751.4915, 777.4700, 795.4831, 913.5570, 957.5609, 1119.5493	Triterpenoid saponin (Eryngioside *C*)	3	TT	-	5
81	11.74	[M − H]^−^	373.09302	373.09235	1.8	C_19_H_18_O_8_	123.0437, 135.0438, 149.0595, 160.0153, 175.0390, 179.0340, 193.0498, 197.0448	Methyl rosmarinate	3	HC	6479915	
82	11.97	[M − H]^−^	285.04071	285.04047	0.8	C_15_H_10_O_6_	68.2225, 164.8401, 171.4909, 175.0386, 285.0380	Luteolin	2	F	5280445	6
83	11.99	[M + H]^+^	303.05045	303.04993	1.7	C_15_H_10_O_7_	144.9594, 160.2895, 303.0504	Quercetin	1^s^	F	5280343	1
	11.99	[M − H]^−^	301.03513	301.03537	−0.8	C_15_H_10_O_7_	151.0019, 178.9968, 215.4516, 243.6472, 301.0371					
84	12.22	[M − H]^−^	1103.52	1103.52744	−6.7	C_53_H_84_O_24_	-	Triterpenoid saponin	3	TT	-	
85	13.63	[M − H]^−^	285.04074	285.04047	0.9	C_15_H_10_O_6_	176.8428, 187.0409, 285.0426	Kaempferol	1^s^	F	5280863	6
86	13.84	[M − H]^−^	209.11772	209.11832	−2.9	C_12_H_18_O_3_	59.0122, 97.0641, 165.1275, 209.1182	Jasmonic acid	2	O	5281166	
87	14.05	[M − H]^−^	161.02324	161.02387	−3.9	C_9_H_6_O_3_	133.0287, 161.0232	Umbelliferone	2	C	5281426	2
	14.05	[M + H]^+^	163.03908	163.03897	0.7	C_9_H_6_O_3_	119.0368, 121.0656, 137.3907, 145.0288, 163.0385					
88	14.87	[M − H]^−^	1117.5357	1117.54309	−6.6	C_54_H_86_O_24_	583.3667, 715.4624, 743.4582, 937.5284, 985.5461, 1027.5735, 1075.5281,	Triterpenoid saponin	3	TT	-	
89	15.2	[M − H]^−^	1203.53925	1203.54348	−3.5	C_57_H_88_O_27_	645.4126, 715.4597, 743.4602, 1099.5226, 1159.5333	Triterpenoid saponin	3	TT	-	
90	15.27	[M − H]^−^	1071.4957	1071.50122	−5.2	C_52_H_80_O_23_	436.7717, 746.7781, 772.1053	Triterpenoid saponin	3	TT	-	
91	15.31	[M − H]^−^	1159.54309	1159.5384	4	C_52_H_88_O_28_	436.1860, 587.3589, 715.4593, 1099.5171	Triterpenoid saponin	3	TT	-	
92	17.3	[M − H]^−^	1099.52958	1099.53252	−2.7	C_54_H_84_O_23_	734.0673, 890.0820	Triterpenoid saponin (Eryngioside J)	3	TT	-	5
93	17.52	[M + H]^+^	177.05464	177.05463	0.1	C_10_H_8_O_3_	93.0346, 119.0863, 121.0391, 135.0801, 145.0289, 177.0549	7-Methoxycoumarin	2	C	5280567	2
94	17.71	[M + H]^+^	969.51023	969.50591	4.5	C_49_H_76_O_19_	-	Triterpenoid saponin (3-*O*–β-d-glucopyranosyl-(1 → 2)-β-d glucuronopyranosyl- 22-*O*-angeloyl-R1-barrigenol)	3	TT	-	7
95	18.22	[M − H]^−^	1187.54376	1187.54857	−4	C_57_H_88_O_26_	-	Triterpenoid saponin	3	TT	-	
96	18.31	[M + H]^+^	911.50453	911.50043	4.5	C_47_H_74_O_17_	-	Triterpenoid saponin (3-*O*-β-dglucopyranosyl-(1 → 2)-β-d-glucuronopyranosyl-22-*O*-angeloyl-A1- barrigenol)	3	TT	-	7
97	18.4	[M − H]^−^	1041.58291	1041.57868	4.1	C_57_H_86_O_17_	489.3563, 502.9471, 583.3742, 603.3950, 639.4351,	Triterpenoid saponin	3	TT	-	
98	21.07	[M + H]^+^	293.2114	293.21112	1	C_18_H_28_O_3_	-	OPDA	3	O	656750	
99	23.01	[M − H]^−^	471.34903	471.34799	2.2	C_30_H_48_O_4_	-	Triterpenoid sapogenin	3	TT	-	

^a^ Metabolite identification level according to Metabolite Standards Initiative recommendation (1—identified metabolites, 2—putatively annotated compounds, 3—putatively characterized compound classes, 4—unknown compounds); ^b^ Group of compounds: AA—amino acids, BA—benzoic acid derivates, C—coumarins, HC——hydroxycinnamic acid derivates, F—flavonoids, N—nucleotide, TT—triterpenoids, O—other compounds; ^c^ CID—Compound ID, PubChem; ^s^ Compounds identified with using commercial standards.

**Table 3:** The effect of the extract from shoots from in vivo plant of *Eryngium alpinum* L. [0.5 mg/mL, 2.5 mg/mL, 5 mg/mL] on inhibition of *Acanthamoeba* trophozoites during four days of treatment.

Extract Concentration [mg/mL]	Duration of Treatment [Days]		
	2nd Day	3rd Day	4th Day
	MN ± SD	MN ± SD	MN ± SD
control	5.62 ± 1.96	11.93 ± 2.33	17.06 ± 2.95
0.5	5.00 ± 2.26	11.83 ± 2.73	17.06 ± 2.50
2.5	4.62 ± 1.50	11.54 ± 3.66	16.44 ± 6.61
5	2.67 ± 1.88 *	5.33 ± 3.98 *	6.69 ± 3.08 *

MN—mean number of trophozoites. * *p* < 0.05 statistically significant difference in comparison with the control during the same time interval; *n* = 18.

**Table 4:** The effect of the extract from in vitro shoot cultures of *Eryngium alpinum* L. [0.5 mg/mL, 2.5 mg/mL, 5 mg/mL] on inhibition of *Acanthamoeba* trophozoites during four days of treatment.

Extract Concentration [mg/mL]	Duration of Treatment [Days]		
	2nd Day	3rd Day	4th Day
	MN ± SD	MN ± SD	MN ± SD
control	5.62 ± 1.96	11.53 ± 2.33	17.06 ± 2.95
0.5	2.53 ± 1.88 *	7.46 ± 3.00 *	13.45 ± 3.58 *
2.5	1.89 ± 1.37 *	6.89 ± 2.33 *	12.69 ± 3.46 *
5	1.06 ± 1.35 *	3.76 ± 2.31 *	5.57 ± 2.02 *

MN—mean number of trophozoites. * *p* < 0.05 statistically significant difference in comparison with the control during the same time interval; *n* = 18.

**Table 5:** Determination of IC_50_ [mg/mL] for the studied extracts of *Eryngium alpinum* L.

Plant Material	IC_50_ 2nd Day	IC_50_ 3rd Day	IC_50_ 4th Day
Shoots from in vivo plants	4.80 mg/ml	4.80 mg/ml	4.60 mg/ml
Shoots from in vitro cultures	0.35 mg/ml	3.50 mg/ml	4.15 mg/ml
